# Impact of fermented rapeseed cake mixed *Bacillus velezensis* on the bacterial community structure and cultivation of tobacco cultivar K326

**DOI:** 10.1038/s41598-025-08400-9

**Published:** 2025-07-10

**Authors:** Yongjin Liang, Mingxing Zhang, Bo Peng, Xiangnan Zeng, Xin Li, Jianyu Wei

**Affiliations:** 1 China Tobacco Guangxi Industrial CO. LTD., Guangxi, 530000 China; 2 Hunan Vegetable Research Institute, Changsha, 410011 China; 3 Hunan Engineering Research Center on Excavation and Utilization of the Endophytic Microbial Resources of Plants, Changsha, 410011 China

**Keywords:** Bacterial organic fertilizer, *Bacillus velezensis*, Tobacco cultivar, Rhizosphere, Endophytic, Microbiology, Applied microbiology, Microbial communities, Environmental microbiology

## Abstract

The bacteria-based biofertilizers were widely applied in the tobacco planting. In the present study, the changes of rhizosphere/endophytic bacteria and the chemical components of tobacco k326 was investigated after *Bacillus velezensis* - based biofertilizer (BOF) application treated on three levels, i.e., 30 kg/mu (B30), 60 kg/mu (B60), 90 kg/mu (B90). The BOF treatments affected the bacterial community structure of rhizosphere, root and leaves of tobacco (*p* < 0.05). Noticeably, BOF treatments significantly increased soil available nutrients (AN, AP, AK), and also improved the content of the tested chemicals of k326 to standard levels. The BOF-induced increasing bacterial genera might explain the changes of soil nutrients and chemical constitutions of tobacco leaves, which were *Marmoricola*,* Sphingomonas*,* Bacillus*, and *Pseudomonas*, etc., both with relative higher abundance in B60&B90. By correlation analysis, we found the significant links between soil and foliar chemical components and the identified modules from rhizosphere or endophytes, which included most of the BOF-induced highly-abundant genera herein. All these findings concluded that BOF application increased the beneficial microbes, which could improve soil fertility, thereby promoting tobacco growth and nutrient accumulation in balance. Summary, we suggested that BOF application could be a promising way to achieve the sustainable development of the tobacco industry.

**Supplementary Information:**

The online version contains supplementary material available at 10.1038/s41598-025-08400-9.

## Introduction

Tobacco (*Nicotiana tabacum* L.) is an important cash crop in many parts of the world owing to its high economic value and is one of the most important economic crops in China^1^. The tobacco leaves can be used in many applications such as in cigarette, medical and biofuel industries^2^. However, Tobacco production requires large amounts of synthetic chemical fertilizers and pesticides^3^. These increased soil contaminations such as residual pesticides, and damaged the soil microbial environment and further led soil to be less capable to provide nutrition, which resulted in crops more prone to diseases. All these unfavorable circumstances resulted in serious implications for tobacco quality.

The tobacco leaves quality is mainly influenced by the climate, soil properties, and microorganism as the climatic factors were reported to be the main factor influencing the tobacco quality. Further, Tobacco smoking characteristics are greatly depended on a variety of metabolic substances in leaves and their proportions^4^. It is nearly impossible to upgrade the tobacco quality by changing the environmental conditions on a broad scale, but it can be effectively improved by changing the soil properties and microbiota^5,6^. Moreover, it is worth noting that microorganism broadly existed in and out plants, and these colonized in rhizosphere zones can improve soil nutrient cycling^7,8^, while others coevolving with the host plant may affect the biochemical processing of metabolism within host plants^9,10^. Therefore, it is significant to investigate the varied changes of the microbial component structures and their diversity functions along with the planting time.

The improvement of Tobacco quality starts with the farmland preparation and field management, especially fertilizer application control. Recently, a number of newly developed fertilizers based on organic materials and beneficial microbes became hotspots in fertilizer industry for their ecofriendly and helpful influences. Organic materials primarily originate from agricultural byproducts, including plant-derived sources (e.g., rapeseed cakes, straw, bean residues) and animal-derived sources (e.g., livestock manure). Potential hazards exist in animal-derived fertilizers, including heavy metal and antibiotic contamination. In contrast, rapeseed cake is a nitrogen-rich organic fertilizer with high organic matter content. After fermentation, it produces organic acids and humus, demonstrating excellent soil amelioration properties. And varieties of PGPR have been studied and coupled with fertilizers applied into field productions, including the species *Pseudomonas*,* Bacillus*,* Enterobacter*,* Klebsiella*,* Azobacter*,* Variovorax*,* Azosprillum*, and *Serratia*^11^. Notably, Bacillus velezensis, a functional strain with spore-forming ability, exhibits strong survival under stress conditions like drought and high temperatures, ensuring field application persistence. Its multifunctional traits (e.g., nutrient solubilization and pathogen suppression) reduce chemical input reliance, offering greater agricultural cost-effectiveness than single-function biofertilizers. Likewise, application of biofertilizers is known as a sustainable solution for reducing and eliminating the chemical inputs in sustainable agricultural systems^12^. Numerous researches noted that the combined application of bio-organic fertilizer and chemical fertilizer can increase the soil enzyme activity^13^, improve the soil fertility^14^, and inhibit the pathogens incidences of soils^15^. Others reported the significant effects of the biofertilizer addition on yields and quality in maize^8^, pear^16^, Panax ginseng^12^, Mandarin fruit^17^. Numerous field experiments have been conducted to assess the effectiveness of these biofertilizers in major tobacco-growing regions. These studies focus on understanding their nutrient release patterns, adaptability to local conditions, and biocontrol efficacy evaluation^18^. For example, rapeseed cake organic fertilizer (inoculated with *Bacillus thuringiensis*) was tested in central China^19^, dreg bean organic fertilizer in north China^20^, and tobacco-specific organic base fertilizer (containing *Burkholderia* and *Purpureocillium lilacinum*) in southwestern China^21^, among others. And a series of the invention patent on organic fertilizer and functional microorganisms were applied for^22,23^. The application of BOFs in tobacco farming represents a novel approach to sustainable agriculture, as it integrates waste recycling, microbial biotechnology, and precision fertilization. Domestic attention to bio-organic fertilizer development and application will promote sustainable and eco-friendly tobacco production.

It has been noted that soil properties have distinct effects on the biomass and structure of soil microbes^24^, as well as the quality and yield of tobacco^25^. the chemical compositions of tobacco leaves correlates with the major microbial physiological groups in the rhizosphere soils^4^. The functional microorganisms played key roles among the physical and biochemical courses within and without host plants. However, the understanding of specific microbial species affecting the quality of tobacco leaves in specific natural environment is still not clear. Therefore, it is of utmost priority to certify the key species related to tobacco quality, which serves as the foundation of exploring microbial resources for tobacco quality improvement in the Xingning tobacco-growing area in western Hunan Province, China. K326 is a flue-cured tobacco variety known for its robust aroma and slender leaf morphology. The quality of its cured leaves is highly dependent on a balanced and adequate supply of soil nutrients, as well as favorable climatic conditions. The biofertilizer addition into Tobacco fields were scarcely reported about the effects on Tobacco microbiota structures and leaves chemical components. In the present study, we utilized the purchased organic fertilizer (fermentation from colza cake), mixed with our lab own a functional species of *Bacillus velezensis* with 5% (w: w), which was as the candidate biofertilizer applying into soils before 10 days of Tobacco seedlings transplanting. The objectives of the study were as follows: (1) to evaluate the extent to which biofertilizer addition can change microbial structure composition and diversity in tobacco rhizospheric and endophytic zones. (2) to assess the impact of biofertilizer application on the improvement in Tobacco quality and (3) to explore the potential mechanisms that induce changes in microbial diversity and Tobacco quality.

## Results

### Soil and tobacco leaf nutrient analysis

**Fig. 1:**
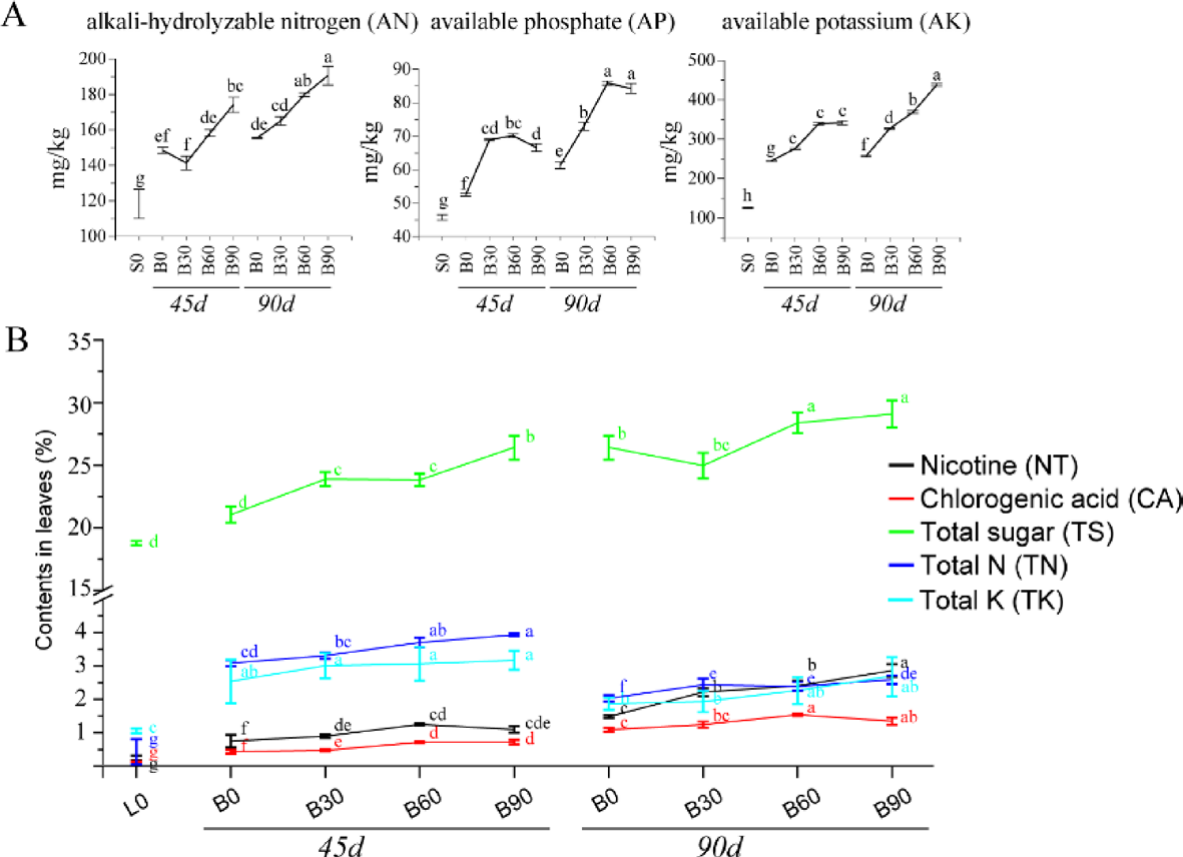
Changes of soil available nutrient contents (**A**) and the five key chemical compositions (**B**) of middle leaves after transplanting days of 0d, 45d, 90d under biofertilizer treatments.

Before the biofertilizer treatment (S0), soil available nutrients maintained medium levels, that is 118.27 mg/kg for AN (alkali-hydrolyzed nitrogen), 45.75 mg/kg for AP (available phosphate), 155.89 mg/kg for AK (available potassium) (Fig. 1A). For the two sampling dates at 45d and 90d, there both were significant differences of the tested chemical contents in soils and leaves between conventional chemical fertilizer group (B0) and biofertilizer treatments (B30, B60, B90). In soils, compared to B0, bio-treatments significantly improved soil AN, AP, AK contents in Fig. 1. For example, bio-treatments (B60, B90) raised AN content by 6.5-22.6%, AP by 26.86-40.15%, AK by 38.3-70.3%. And evidently, B90 improved AK content in soils to 342.01 mg/kg (45d) and 438.24 mg/kg (90d) to maintain high level available K nutrient for tobacco development. The soil available nutrients under the 90 kg/mu biofertilizer application (B90) looked significantly higher than B30 and B60 treatments.

The chemical compounds in the course of the Tobacco were detected (Fig. 1B), that is total nitrogen (TN), total sugar (TS), nicotine (NT), chlorogenic acid (CA) and Total K (TK). The results showed that contents of TS, TN and TK were markedly higher in booming period (45d) than in seedling period. At 45d, the samples of B0 (chemical fertilizer application) and B30 (biofertilizer application) had similar levels of the five tested chemicals, and the contents of TN and TS in B0&B30 were lower to the higher bio-treatment (B90). For keeping nutrients in Tobacco leaves from losing to flower, topping measures for the flower must be taken at around 60d. It could be seen that the chemical components in leaves amassed distinctively after that measure, such as TS and NT at 90d, however the contrary tendency of the contents of TN and TK. Generally, low biofertilizer (B30) improved the contents of Tobacco’s chemical substances, which were not greater than high biofertilization effects (B60, B90) when compared with B0. The B60&B90 increased the levels of TS, NT, TN and TK, which were accord with the standard levels for making the high-quality tobacco products (Table S3). Of course, the production of high-quality tobacco would rely on both appearance traits of leaves and the overall balance of the chemical compounds of leaves, included the tested matters, as well as other ingredients of starch, protein, Cl, etc.

### Microbial diversity and community composition

**Fig. 2:**
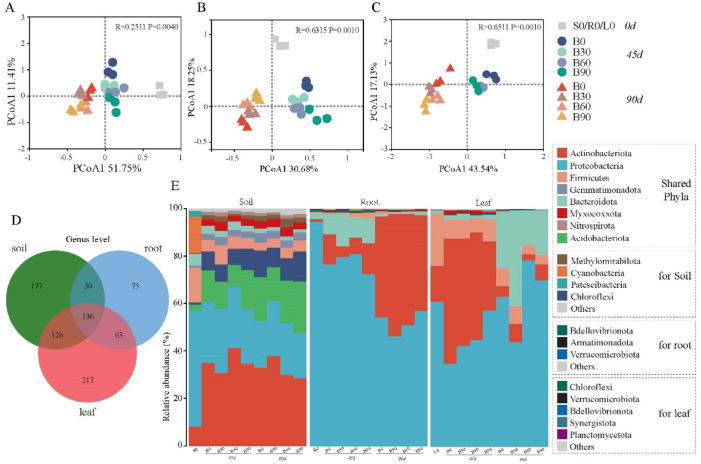
Principal co-ordinates analysis of microbial community (**A**–**C**) and relative abundance of bacterial community (**E**) at phylum levels from tobacco three habitats (i.e., rhizosphere, root, leaves), and their genus-level venn diagram analysis (**D**) under biofertilizer treatments.

In comparison to the conventional fertilizer application (B0), the bio-treatments had higher values in the bacterial community diversity of the rhizosphere, root and leaves, but there were little differences of the ACE richness indices among the two groups (Fig. S1). The principal component (PCoA) analysis indicated that the bacterial constitution of three habitats before transplanting were apparently different from the samples afterwards found at the first axis. And there were markedly dissimilarities in bacterial constitutions from 45d to 90d (Fig. 2). In the same sampling dates (i.e., 45d, 90d), it saw high similarities for bacterial community either in rhizopheric soils or in endogenous root and leaves habitats.

This constitution differences also could be seen at in bacterial phylum relative abundance levels. The soils (S0) enriched in bacterial phyla of Protcobacteria, Firmicutes, Cyanobacteria, were different from the samples from the growth stages (Fig. 2E). At the periods of fast growing and maturation of Tobacco, there were highly similar components for the dominant bacterial phyla members, such as Actinobacteria, Protcobacteria, Acidobacteria, etc. For endogenous bacteria groups in root and leaves samples, the most phyla were Protcobacteria, Actinobacteria, relative abundances of the two from 51.3 to 95.7% across all samples. And the two phyla showed totally inverse changing trends in their abundances from the 45th day to the 90th day. The less abundant phyla were Bacteroidota in roots and Firmicutes in leaves. The relative abundance of phyla between chemical fertilizer group (B0) and bacterial fertilization groups (B30, B60, B90) had more dissimilarity than that among three biofertilization treatments. Moreover, the changes of relative abundance for some bacterial phyla were accordance with the biofertilizer treated levels (Fig. 2).

**Fig. 3:**
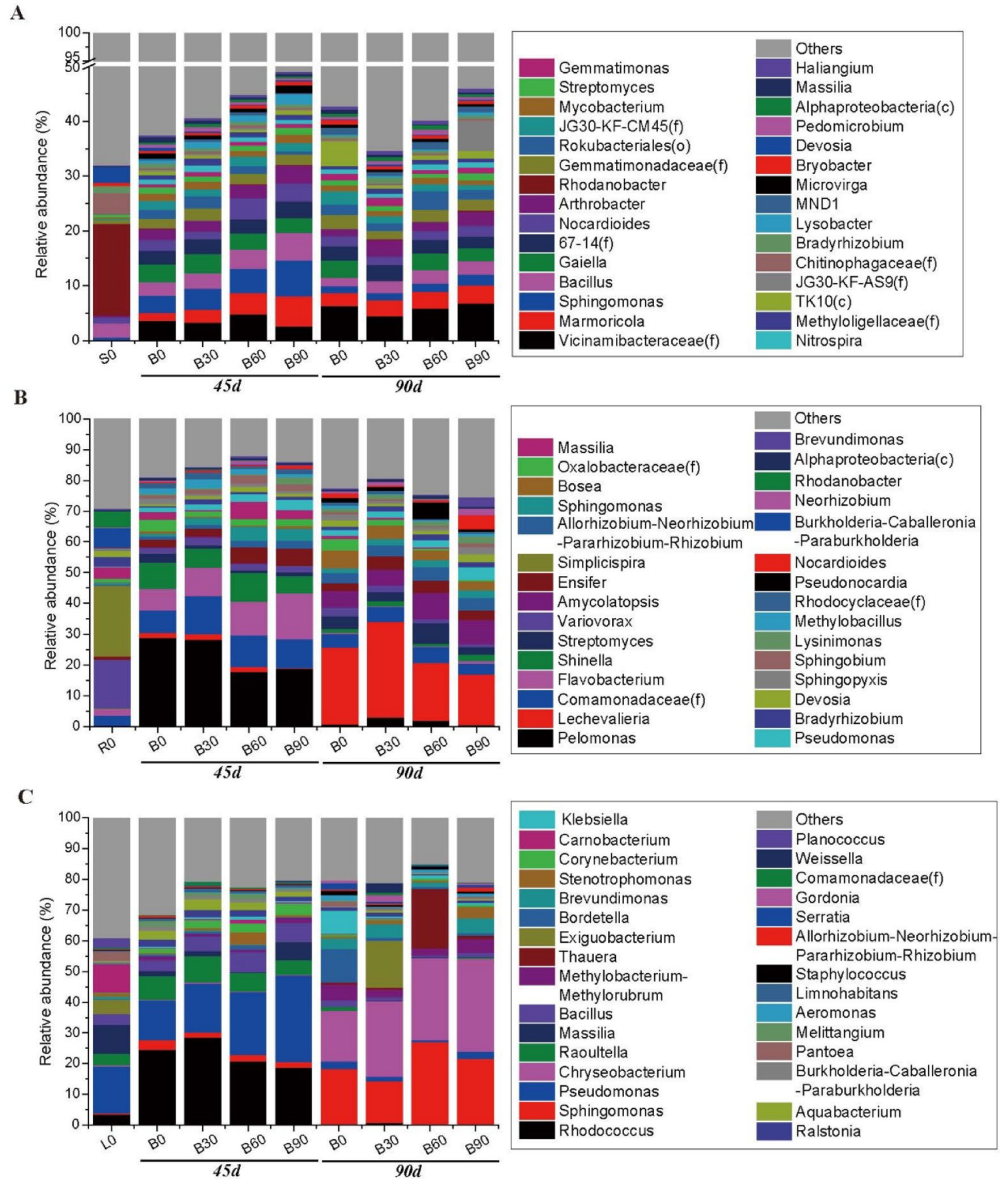
Distribution barplot of bacterial community of top 30 genera from biofertilizer treated samples of Tobacco rhizosphere (**A**), root (**B**), leaves (**C**) at three sampling times.

Tobacco three habitats (i.e., rhizosphere, root and leaves) exhibited a diversity of bacterial genera in venn diagram. The sharing genera among three bacterial communities were 136, the specific-owning were 177 in soils and 217 in leaves (Fig. 2D). We calculated the relative abundance of the top 30 genera, which accounted with the range of 32–45% in soil bacterial community, while 70–88% in roots and 61–85% in leaves (Fig. 3). There were some soil bacterial genera that changed their relative abundance positively followed with biofertilizer addition levels (Table S4), such as *Marmoricola*, *Sphingomonas*, *Lysobacter* (Proteobacteria), *Nocardioides* (Actinobacteriota), *Bacillus* (Firmicutes), *Haliangium* (Myxococcota). For endogenous bacterial groups in the course of experiments, the genera accordant with the biofertilization application levels were also found (Table S4), such as *Flavobacterium* (Bacteroidota), *Allorhizobium-Neorhizobium-Pararhizobium-Rhizobium*, *Pseudomonas*, *Sphingobium* (Proteobacteria) in root samples, and *Bacillus* (Firmicutes), *Sphingomonas*, *Pseudomonas* (Proteobacteria), *Chryseobacterium* (Bacteroidota), *Corynebacterium* (Actinobacteriota) in leaves samples. Among these varied rhizosphere and endogenous bacterial species, the most significant differences were found between growth stages rather than treatment measures (Table 1).

**Table 1:** Results of the permutational analysis of variance, showing R^2^ and significance of the treat and growth stage factors for the community composition of bacteria of tobacco parts.

Effect factors	Soil bacterial community		Root bacterial community		Leaf bacterial community	
	R^2^	*P*-value	R^2^	*P*-value	R^2^	*P*-value
treat	0.5517	0.001***	0.2346	0.023*	0.1888	0.0407*
stage	0.5526	0.001***	0.4729	0.001***	0.5443	0.001***

*p*-values are significant below 0.05: *p < 0.05, ***p < 0.001.

### Microbial co-occurrence patterns driven by bio-fertilizers

**Fig. 4:**
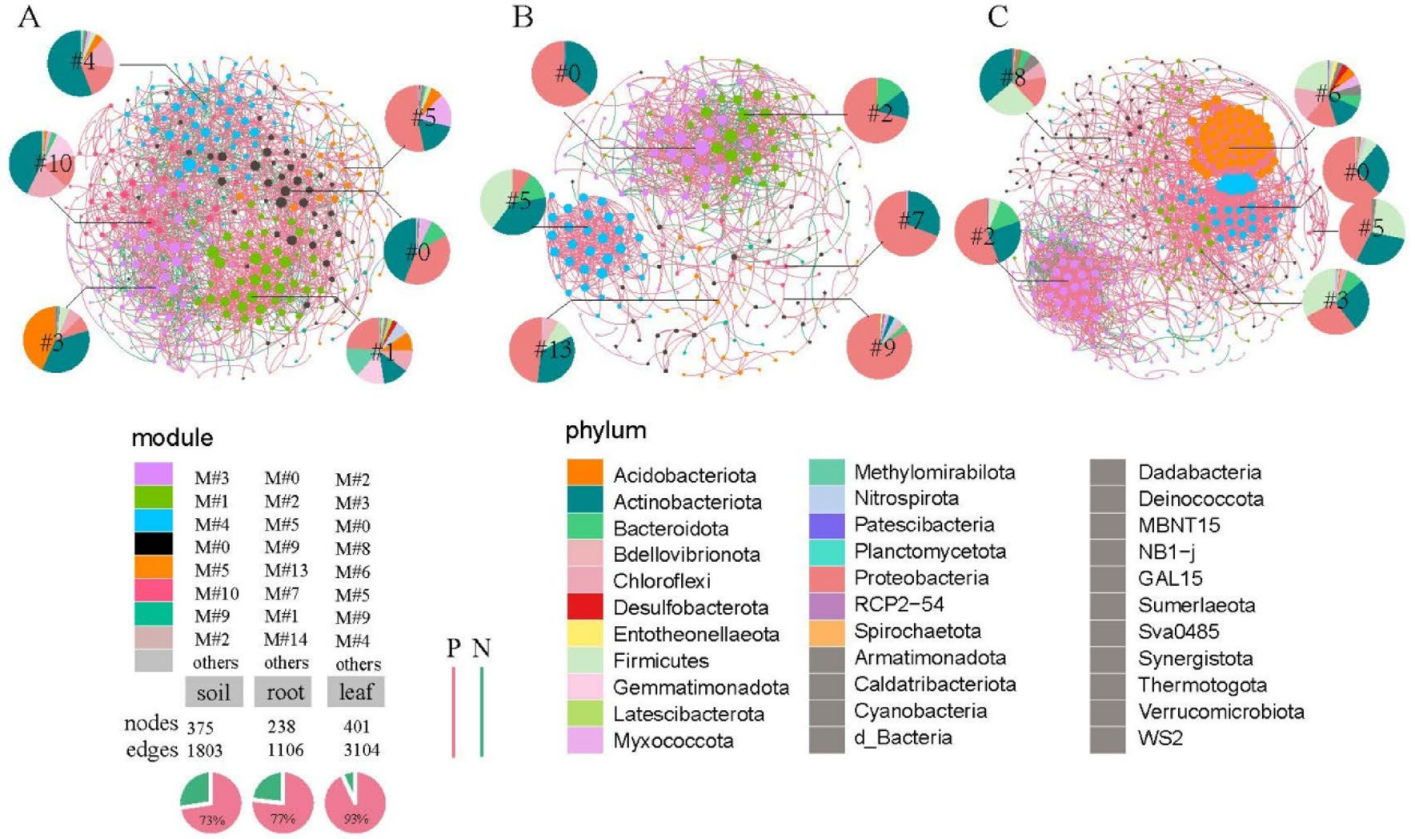
Co-occurrence network analysis of bacterial community (genus level) from tobacco rhizosphere (**A**), root (**B**), leaves (**C**) habitats. The phylum-level relative abundances of their belonging modules were counted. Circles represent individual bacterial genus and are colored according to their Phylum assignment.

Further, we explored the extent to which biofertilizer regimes impacted co-occurrence patterns of microbial communities in different biological sites. The degrees of all three networks followed power-law distributions (Fig. S2), indicating their scale-free feature and non-random co-occurrence patterns. By building their respective identically sized random networks, we compared the values of average path length (APL), average clustering coefficient (ACC), and modularity index (MI) with in empirical networks (Table S5), the higher values suggesting that the empirical networks had prominent ‘small-world’ modularity and hierarchy of their topological properties. And modularity values were higher than the random networks and greater than 0.4, indicating that the constructed networks had modular structures (Table S5). The root bacteria network comprised the lowest number of significantly cooccurring genera (1016 edges) and was the least complex, comparing to soil and leaves networks. And average path length and clustering coefficient in foliar network were significantly higher than the other two networks, which suggests the complexity of the foliar bacterial community (Table S5). Interestingly, the interconnected networks of soil, root and leaves both showed highly positive relationships that accounted for 73%, 77%, 93% of total edges, respectively (Fig. 4).

Of the nodes in networks, the one with Zi ≥ 2.5 or Pi ≥ 0.62 were determined as the keystone species. The three biological networks both harbored a number of connectors (Zi < 2.5&Pi > 0.62), that is 109 (29%), 56 (23.5%), 72 (18%), respectively (Table S7). The highly abundant connectors were 67 − 14 (f), *Nocardioides*,* Micromonospora*,* Paenibacillus*,* Xanthobacteraceae* (f), *Mycobacterium* in rhizosphere; *Streptomyces*,* Sphingomonas*,* Pseudomonas*,* Bacillus*,* Agromyces* in root; *Massilia*, *Stenotrophomonas*, *Bordetella*, *Weissella*, *Lactococcus*, *Lysinimonas* in leaf tissues. A few module hubs such as *Candidatus_Solibacter*, *Methyloligellaceae* (f), *Haliangium*, and *Xanthobacteraceae* (f), *Nocardioides* were found in the rhizosphere and root networks. These important connectors variedly existed in networks modules, such as belonging to M#0, M#3, M#5 and M#10 in the rhizosphere network, to M#0, M#9, M#13 in roots, and to M#0, M#2, M#5, M#8 in the leaves (Table S6). Bio-fertilization addition resulted in drastic changes in the relative abundance within modules. In the rhizosphere network, the relative abundance of M#0, M#1, M#3, M#4, M#5 was strongly changed among samples (Fig. 4), particularly increasing under high-levels biofertilizer additions, such as M#0, M#5, M#10. Similar changes were found in endogenous niches that high biofertilizer treatment triggered the increasing of the relative abundance of M#0, M#2, M#9 in the foliar network (Fig. 4).

### Prediction of bacterial community functions

**Fig. 5:**
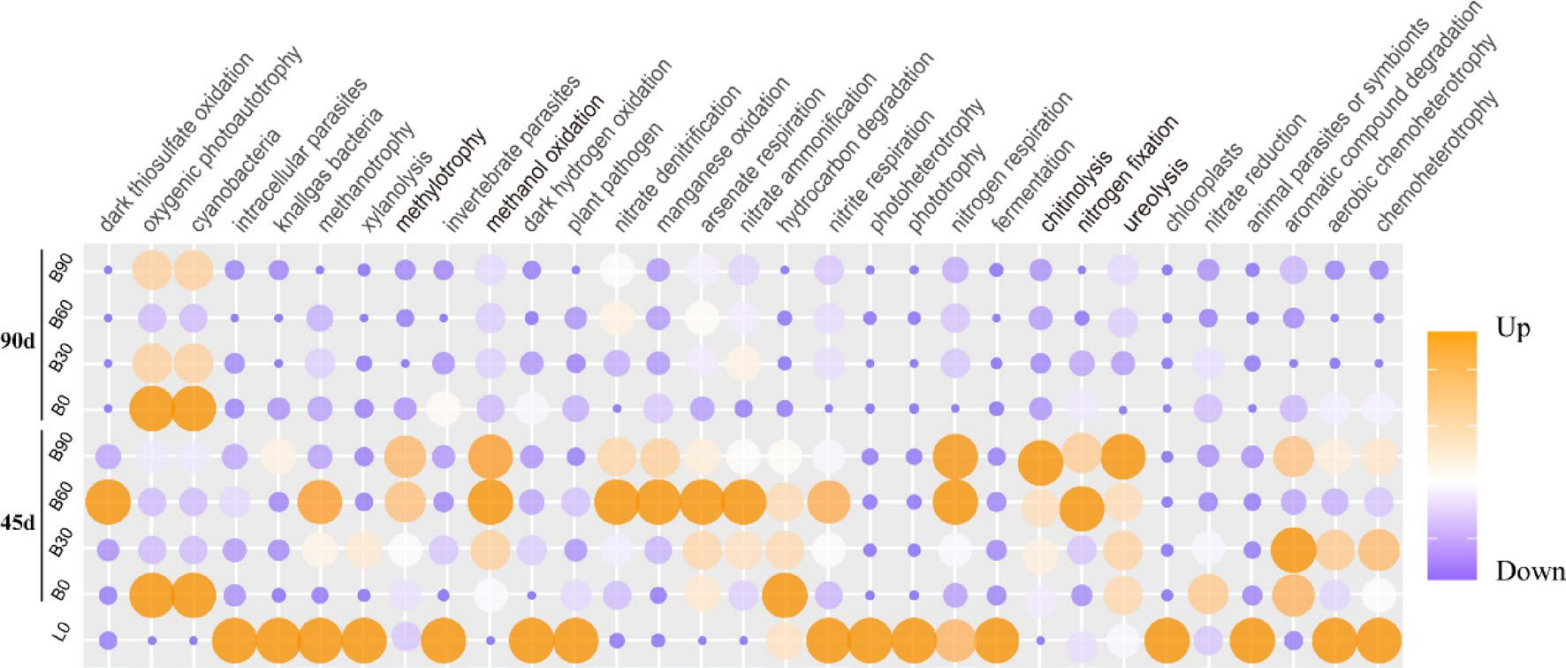
Functional annotations of rhizosphere bacterial community by FAPROTAX database. Bubble heatmap presented scale values of the abundances of the bacterial community.

**Fig. 6:**
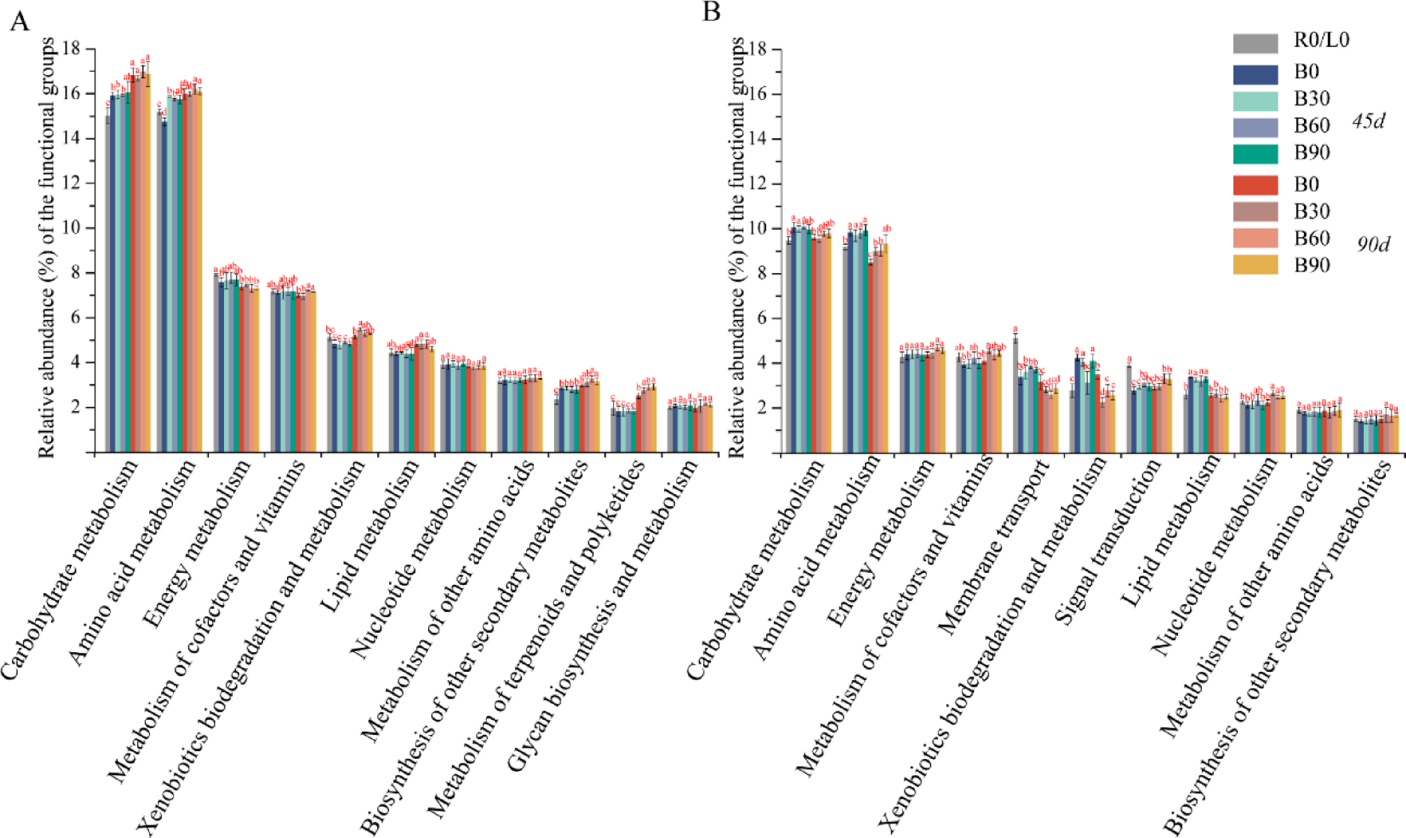
Functional annotations of endophytes (**A**, root;** B**, leaves) predicted by PICRUST2. 12 bio-chemical metabolic pathways of KEGG level-2 pathways were selected for further analysis.

Biofertilizer treatment triggered changes of microbial community structure, as well as the relative abundances for the functional groups in habitats. According to Fig. 6, some N, C cycling processes varied obviously among the sampling sites. There were 13 functional groups with their relative abundance declining in the course of Tobacco’s growth than that of the unplanted soil. Moreover, microbes related to the functions of nitrate denitrification, nitrate ammonification, chitinolysis, ureolysis, aromatic compound degradation were more abundant at 45d than at 90d. Compared to B0 at 45d, high-biofertilizer treatments (B60&B90) induced the increasing of the N-cycling functional groups, such as chitinolysis, ureolysis, nitrogen respiration (Fig. 5). When Tobacco’s leaves maturity at 90d, the soil functional microbes overall kept relative low abundance than the early stages.

PICRUST was used to predict the relative abundances of microbe functional genes at KEGG level2 (Fig. 6). The most abundant pathways in all samples were amino acid metabolism and carbohydrate metabolism, followed by energy metabolism, and metabolism of cofactors and vitamins both in bacterial community of root and leaves. In general, the functional abundance of all the 11 pathways were found higher in root than in leaves of tobacco. Besides, the relative abundance of some metabolism pathways was increased by the biotreatments (B30-B90) in Fig. 6. Overall, the differences of these metabolic functional abundances from the development stages were greater than the treatments, as the results showed the permutational analysis of effects of the treat and growth stage factors on the bacterial community composition of Tobacco rhizosphere, root, leaves (Table 1).

### Correlation between bacteria groups and nutritional variables

**Fig. 7:**
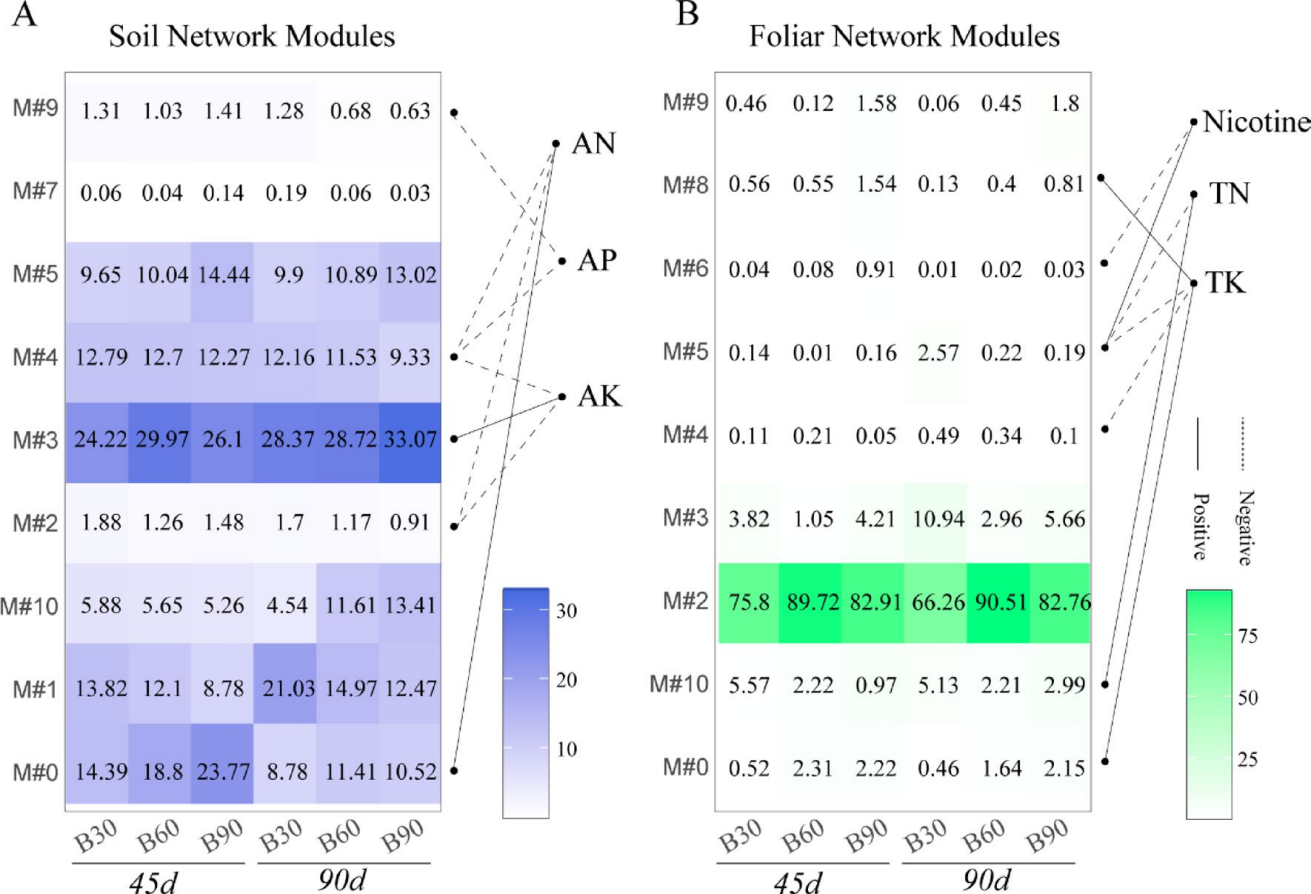
Heatmap shown the relative abundance of the key modules from rhizosphere (**A**) and foliar interactive network (**B**). The correlations were calculated between the network modules and soil physiochemical properties or foliar chemical contents.

In this study, we further explored the correlations between Tobacco’s rhizospheric bacterial network modules and soil physiochemical properties, to search important functional groups for soil nutritional cycling. In Fig. 7, three soil edaphic factors of AN, AP and AK were found the correlations with modularity groups in soil bacterial network and root bacterial network. The relative abundance of M#0 was positively related to the changes of AN, which there were more higher abundances of M#0 in 45d treatments than that of 90d. The M#0 had 12 the keystone species (connectors) in the rhizosphere co-occurrence network, which were *Nocardioides* (11357), *Pajaroellobacter* (423), *Amycolatopsis* (262), f_*Blastocatellaceae* (240), *Solibacillus* (219), f_*Nocardioidaceae* (202), *TM7a* (189), etc. (Table S6, Table S7). In the context of bacterial genus, soil AN was significantly positively correlated with *Nocardioides* (0.75), *Bacillus* (0.75), *Marmoricola* (0.73), and *Sphingomonas* (0.69) (Fig. S4). Similarly, the M#3 with the enriched genera of f_*67 − 14* (13961), *Kribbella* (1552), *Paenibacillus* (1115), *Hydrogenispora* (1089) were significantly positive to soil AK content, and it also were found a higher relative abundance in B90 treatment both at 45d and 90d (Fig. 7A, Table S7). There was a significant positive correlation between the relative abundance of *Pseudomonas* (0.82), *Paenibacillus* (0.78), *Massilia* (0.77), f_*67 − 14* (0.71) and the AK content in the soil.

Further, we tried to find the functional clusters in foliar network affecting Tobacco’s chemical contents. According to the correlation analysis, the chemical components of NT, TN, TK were positively related to the foliar network modules M#0, M#5, M#8&M#10 accordingly (Fig. 7B), which harbored keystone species with number of 16, 5, 17, 2, respectively. However, M#2 with a markedly-high abundance, it was no significant links found with the foliar chemical compounds. At the genus level, correlation analysis revealed that the relative abundance of the bacterial genera is associated with changes in the content of chemical components. It can be observed that the genus *Pseudomonas*, *Bacillus* showed a significant positive correlation with both TN and TK. In addition, 8 bacterial genera (*Chryseobacterium*, *Lechevalieria*, etc.) correlated positively with TS, and 4 genera (*Klebsiella*, *Comamonas*, *Aureimonas*, *Lactococcus*) with NT (Fig. S4).

## Discussion

*Bacillus* is a significant type of plant growth-promoting rhizobacteria (PGPR), widely present in agricultural soils^11^. Research has shown that *Bacillus* species plays a notable role in nutrient cycling, promoting plant growth, and disease resistance, benefiting both soil and plants. Currently commercialized *Bacillus* strains, such as *B. subtilis*, *B. amyloliquefaciens*, and *B. velezensis*, are formulated into organic fertilizers. Their effects have been extensively tested in agricultural crops^6,26,27^, in different soil types^28,29^ and cropping systems^30^. In this study, we applied the biofertilizer of rapeseed cake compounded with *Bacillus subtilis* into tobacco field. The bio-treatments (B30-B90) did not significantly enhance bacterial diversity in soil, roots, or leaves compared to chemical fertilizer. Additionally, the ACE indices of rhizosphere bacteria at 45 days and root bacteria at 90 days were lower in the treatment groups than in the control. This may be due to microbial organic fertilizer enhancing specific functional bacteria while reducing overall rhizobacterial diversity^8^. Through permutational analysis of biofertilizer effects on the rhizosphere, root and leaves bacterial community, we found the significant effects of BOF treatments with the p-value of 0.001*** (soil), 0.023* (root), 0.0407* (leaf), which were in lined with the reported studies^12^. Overall, the differences in the bacterial community influenced by developmental stages were significantly greater than those caused by treatment measures. (Table 1). Bei et al. (2018) also reported a similar view, stating that sampling time had a much greater influence on the soil microbiome than the fertilizer regime^31^.

The composition differences at the phylum level in the rhizosphere soil bacterial communities were relatively minor among different treatments (chemical fertilizer or bio-organic fertilizer). The bacterial phyla of Actinobacteriota and Proteobacteria were taken a most majority from 52 to 60% of all biotreatments. This is consistent with the findings of previous studies, such as the application of Stanley compound fertilizer (containing *B. licheniformis* or *B. amyloliquefaciens*) in maize crops^8^, the use of organic fertilizers (e.g., straw and livestock manure) or *B. subtilis* treatment in traditional crop cultivation (leafy vegetables and tuber crops)^32^, and the application of bio-fertilizers in tea plantation soils^33^. Indicator microorganisms in different niches provide guidance for agricultural practices. Thus, we also analyzed bacterial communities in tobacco roots and leaves after applying *Bacillus velezensis* rapeseed cake bio-organic fertilizer. There were two dominated bacterial phyla of Proteobacteria and Actinobacteria in tobacco roots, reached up to 83.2–97.4% across the experiments. Still, the less dominated phylum of Bacteroidota was found during the early stages of tobacco growth. With the relative low abundance of Firmicutes (< 5%) from all samples, it is noteworthy that high usage of biofertilizers (B90) significantly increased the relative abundance of Firmicutes. The increasing bacterial phyla in roots in early stages (45d) were partially due to the increased relative abundance of the rizhosphere soils induced by the bio-organic fertilizer as the previous reports. However, the decreasing Proteobacteria could be explained by the niche competition of bacterial community in roots, as well as the balance control of the host tobacco. For foliar bacterial groups, we could see the obviously changes at phylum level within 45d after fertilizer application. Similarly, the top two bacterial phyla (Proteobacteria and Actinobacteria) in leaves were of large proportions as in the roots. B90 treatment significantly increased the Proteobacteria both in the times of 45d and 90d.

The healthy root of tobacco plant harbored the abundant bacteria genus of *Pseudomonas*, *Streptomyces*, *Bacillus*, *Lysobacter*, and *Paenibacillus*^34^, which were not always according with our results, which might due to the differences of crops^26^, soil types^28,30^, and natural environment. According to Cai et al. reports^35^, the tobacco K326 in seeds were dominated with the bacterial genera of *Pseudomonas*, *Bacillus*, *Sphingomonas*, etc. Similarly, these genera were also found in our analysis as the dominants. Besides, the biofertilizer treatment (higher levels) significantly improved the relative abundance of *Marmoricola*, *Bacillus*, *Nocardioides*, *Sphingomonas*, *Lysobacter* in soils, and *Flavobacterium*, *Sphingobium*, *Ensifer*, *Pseudomonas* in roots. These bacterial genera were reported as positive responders to the bio-organic fertilizer. For instance, *B. velezensis* SQR9 inoculation increased the abundance of *Pseudomonas* and *Lysobacter* in cucumber rhizosphere soil, with *B. velezensis* SQR9 and *Pseudomonas stutzeri* demonstrating synergistic effects^9^. Similarly, field trials using a *B. amyloliquefaciens* W19-enriched bio-organic fertilizer revealed that *Bacillus* amendment stimulated indigenous *Pseudomonas* populations, enhancing banana resistance to *Fusarium* wilt^15^. Additionally, the combination of sheep manure organic fertilizer and *B. subtilis* promoted the growth of *Flavobacterium* and *Sphingomonas* in pineapple rhizosphere soil^36^, while commercial microbial fertilizers were shown to increase the abundance of *Bacillus* and *Pseudomonas* in maize rhizosphere soils^37^. Interestingly, in the early stages of Tobacco (at 45d), the increasing tendency of the endogenous *Pseudomonas* were found in leaves, that is 15.87% (B30), 20.56% (B60), 28.21% (B90), which might be affected by the rhizosphere bio-fertilizer application. Different organic fertilizers or microbial inoculants distinctly shape rhizosphere soil and plant endophytic bacterial communities, demonstrating niche-specific enrichment of functional microbes. This selective enrichment reflects microbial adaptation to fertilization regimes, with community shifts closely linked to organic matter type, microbial traits, and environmental interactions.

In co-occurrence networks analysis, the BOF addition caused a relative highly positive correlations within networks that accounted for 73% in rhizosphere, 77% in root, 93% in leaves orderly, indicating that fertilization may act as bioactive agents and/or enhance synergistic interactions of the soil microbiome, which were in line with the previous study on tea^33^, banana cultivation^38^. Compared to using organic fertilizer or microbial inoculants alone, microbial organic fertilizer (organic carrier + composite functional strains) combines the benefits of both, offering ecological niche complementarity and enhanced functionality. In the short term, the soil microbiome exhibits high sensitivity to organic amendments^33,39^, while the positive impacts on soils are long-term and beneficial for the growth and productivity of crops^31^.

The changes of soil physicochemical properties were reportedly related to the relative abundance of the rhizosphere bacterial genus or their-forming cluster groups^40,41^. de Menezes et al. (2015) reported that the modules built by networks had different relationships to soil variables and concluded that network analysis offers a more comprehensive understanding of microbial group interactions, serving as an effective approach to guide forthcoming research on the ecological functions of soil organisms^40^. We found the relative abundance of module#3 being higher in B90 treatment, were positively linked to soil AK. The module harbored 22 connectors. The bacterial phyla of Acidobacteriota, Actinobacteriota were dominated in the module of soil network. Similarly, the module#0 were correlated to available N content, which enriched in Actinobacteriota and Proteobacteria. The three bacterial phyla generally dominated in the tobacco rhizosphere. Their differently distribution in samples showed influence of biofertilizer application, and that differences in network modules suggested the different degree of interaction of the bacterial genera, or biological choosing. Under BOF application, supplying of CNPK elements to soils could boost these functional bacterial growth and propagation. Within the associating modules of soil network to AN and AK, the key taxa (i.e., connectors) were reported important roles in regulating soil available nutrients. They were *Marmoricola*, *Bacillus*, *Sphingomonas*, *Nocardioides*, *Lysobacter*, f_*Chitinophagaceae*, o_*Vicinamibacterales*, most of them enriched in higher BOF treatments (Table S4). Spearman analysis indicated that some functional bacterial genera in the rhizosphere soil were significantly correlated with soil AN, AP, and AK contents. For example, *Bacillus*, *Pseudomonas* were multifunctional bacteria capable of nitrogen fixation, as well as the mineralization of organic carbon, organic nitrogen, and organic phosphorus^7,11,32^. *Nocardioides* was involved in the nitrogen cycle^42^, *Paenibacillus* possessed the ability to mineralize potassium^11^, and *Pseudomonas* can secrete chitinase, facilitating the decomposition of organic matter in the soil^11^. These functional activities synergistically enhanced nutrient cycling and improve soil fertility. Moreover, the FARPROTAX-based function analysis showed that biotreatments increased the bacterial genera with function of chitinolysis, ureolysis, nitrogen fixation, and C cycling (methanol oxidation, methylotrophy) in the initial stages of growth. This suggests that the application of BOF enriches functional microorganisms, enhances the CNPK cycling processes, and consequently improves soil nutrients, which aligns with the findings of previous studies^7,33^.

As the second genome of plants, plant–associated microorganisms are closely related to plant growth and metabolism^43^. The KEGG database annotated functional abundance of tobacco endophytes, revealing that bio-treatments increased bacterial groups linked to carbohydrate metabolism, amino acid metabolism. Following the correlations between network modules and chemical compounds in leaves, we further identified the core taxa in leaves co-occurrence network, in which included 74 connectors, such as *Bacillus*, *Sphingomonas*, *Pseudomonas*, *Corynebacterium*, *Massilia*, etc., which might be participated in the physiological process of plant host in some ways, thereby influencing the second metabolism synthesis and production. We also observed that *Bacillus*, *Pseudomonas* was positively correlated with contents of total N (TN) and total K (TK) of tobacco leaves, whereas *Bacillus*, *Sphingomonas* correlated positively with total sugar (TS) content. There reported a strong correlation between endophytic bacteria and second metabolites. Qin et al. (2022) found that triterpenoid levels in Schisandraceae plants were significantly correlated with the abundances of endophytic *Rhizobiaceae*, *Bradyrhizobium*, *Bacillus*^44^. The dynamic changes in endophytic bacterial genera (*Sphingomonas*, *Corynebacterium*, *Massilia*) showed an increasing trend during the developmental progression of ginkgo leaves, and these microbial abundance variations may be associated with flavonoid accumulation^45^. Furthermore, *Pseudomonas* and *Sphingomonas* are important endophytic microorganisms that have been identified in various plant species^44,46^. Notably, *Sphingomonas* possesses the capability to synthesize gibberellins^11^. The endophytes could directly synthesize the same or similar physiological active substances as their host, for the genetic reorganization in the evolutionary process of endophytes^47^. Indirectly, endophytes may promote metabolites accumulation of the host plant in the way of the supply of precursor substances, and enzyme and gene expression^43^, which were extensively reported the beneficial genus of *Bacillus*, *Pseudomonas*,* Sphingomonas*,* Massilia* in the documented studies^48–51^. However, current understanding of the effects of the microbiome on host plant secondary metabolites and their mechanism of action is still limited. This study reveals the influence of functional microbial fertilizers on the tobacco endophytic core microbiome, identifying key correlations between endophytic bacteria and leaf components (nicotine, total nitrogen, and potassium), thereby providing a theoretical foundation for optimizing microbial fertilizers and regulating tobacco quality.

In conclusion, our study demonstrated that BOF enhanced soil major nutrient availability, improved tobacco leaf quality, and enriched beneficial microbes. These effects may extend beyond tobacco cultivation, supporting sustainable agricultural practices by reducing dependency on chemical inputs and fostering a more robust soil microbiome for subsequent crops. Future research could further assess the economic benefits of *Bacillus velezensis* bio-organic fertilizer (BOF) in practical agricultural production, including its effects on improving tobacco yield and flue-cured tobacco quality, as well as the cost savings from reducing chemical fertilizer use. Additionally, long-term field experiments could be designed to monitor the dynamic changes in soil microbial communities after BOF application, particularly the sustained impact on beneficial microbes and the long-term effects on soil health and ecosystem functionality. For large-scale commercial implementation, we also recommend further optimizing BOF application based on soil conditions and nutrient requirements of crops, integrating it with reduced chemical fertilizers for cost-effectiveness, and promoting adoption through training and policy support to facilitate scalable use in tobacco farming. In Xingning tobacco-growing area of south-central China, the 60 kg/mu application rate is recommended as a more cost-effective and agronomically rational choice under conditions of moderate soil fertility. The rapeseed cake combined with *Bacillus* species is a promising green biofertilizer, with potential for wider use across agricultural systems.

## Conclusion

Based on the present data, this study demonstrates that application of the *Bacillus velezensis* biofertilizer had positive effect on tobacco (k326 species) development in terms of soil available nutrients and leaves chemical compounds by reshaping bacterial community structure in rhizosphere (*P* < 0.001) and endosphere (*P* < 0.05). BOF addition into soils significantly increased the content of AN and AK, and also improved the key chemical ingredients of leaves to standard levels. These may due to the increasing beneficial bacteria induced by BOF, such as *Bacillus*, *Sphingomonas*, *Pseudomonas* – as reportedly functional species. Moreover, we have identified key network modules and certain bacterial genera that show significant correlations with soil physicochemical properties or chemical components of tobacco leaves. Further investigation employing multi-omics approaches (metabolomics and transcriptomics) will be conducted in the future, to elucidate the metabolic impact mechanisms of these functional bacteria on the chemical components of K326 tobacco leaves.

## Materials and methods

### Experimental design and samplings

The experiment was carried out from Mar. to Oct. in 2022 in Xinning County, western Hunan Province, China (26°43′ N, 110°85 E), with a climate of seasonal temperate semi-humid monsoon zones. The field has been subjected to a rice-tobacco rotation for years. The last-season remaining rice straw were blended into soils before plowed the land, to provide some available nutrient for the soil. The Tobacco planting had the first chemical fertilizer application at the time of transplanting, and the following three fertilization within the first month of the transplanting. The amount of chemical fertilizer at the four times in the course of the Tobacco were seen in Table S1. We set the control experimental group with the conventional fertilizer level (B0). Based on the control experiment, the biofertilizer experimental groups were added the *Bacillus subtilis* microbial fertilizer with 30 kg/mu, 60 kg/mu, 90 kg/mu, named B30, B60, B90 respectively. The tested microbial biofertilizer mainly contained the fermentation-finished rapeseed cake mixed with *Bacillus velezensis* suspension, a bacterial species isolated from tabacco rhizosphere soil. The chemical properties of the mixed fertilizer are presented in Table S2.

Samplings (S0) of rhizosphere soil samples at 0d were collected before the experimental treatment, and the root (R0) and leaves samples (L0) at 0d were obtained when transplanted. We focused on the changes of bacterial community at mid- and late- stages of Tobacco development after fertilizer treatments. Samplings of rhizosphere soil, root, middle leaves samples were executed at 45d, 90d after the transplanting. Altogether, there were a total of 81 samples with triplicates. All samples on each time collection were quickly cleared by sieving or washing, as well as surface sterilizing^52^, and then separated into two equal samples: one stored in a refrigerator for further bacterial community analysis and another dried in 70℃ for chemical components analysis.

### Measurements of soil and tobacco leaves properties

Soil available nutrients like alkali-hydrolyzable nitrogen (AN), available phosphate (AP), available potassium (AK) was measured, as well as soil pH value in three growing dates of Tobacco. Soil pH was measured using a pH meter and a soil-water ratio of 1:5 (w/v) (Mettler-135 Toledo International Inc., China). The AN in soils included ammonium nitrogen (NH^4+^-N) and nitrate nitrogen (NO^3–]^-N) were extracted with 2 M KCl and then measured by semi-micro Kjeldahl 139 digestion^5^. The ultra-violet spectrophotometer (Unico 137 Instrument Co., LTD, WFZUV-4802 H, Shanghai, China) was used to detect the content of AP using NaHCO3 extraction method, and the flame photometer (Sherwood M410, 138 England) to AK using NH4OAc extraction^27^. The powder samples of Tobacco root and leaves from three sampling dates were prepared by 60℃ drying, then smashing, and further were detected the main chemical ingredients, such as total sugar (TS), total nitrogen (TN), nicotine (NT), and chlorogenic acid (CC). The contents of TN and water-soluble sugars (i.e., TS) were measured according as Wang et al. (2012) described by continuous flow method^53^. Hydrochloric acid was used to extract nicotine levels and 95% ethanol was used for chlorogenic acid, the two extractions that then measured by ultraviolet (UV) spectrophotometry^54^.

### DNA extraction and sequence analysis

Total bacterial community DNA from 81 samples of homogenized soils, roots or leaves were extracted using a Fast DNA spin Kit (MP Biomedicals, USA). The extracted DNA was checked on 1% agarose gel, and DNA concentration and purity were determined with NanoDrop 2000 UV-vis spectrophotometer (Thermo Scientific, USA). For comparing the bacterial community between rhizosphere, root and leaves habitats of tobacco after biofertilizer addition, we used the primers 799 F (5′-GTGCCAGCMGCCGCGGTAA‐3′) and 1193R (5′‐CCCCGYCAATTCMTTTRAGT‐3′) for the V5-V7 region amplification across all samples^55^. Each PCR reaction system contained 20 µL mixture, that is sample DNA (10 ng), 5×FastPfu Buffer (4 µL), 2.5 mM dNTPs (2 µL), FastPfu DNA Polymerase (0.4 µL), BSA (0.2 µL), 5 µM of each primer (2 × 0.8 µL), and the rest of ddH2O. Cycling conditions were described as in Ibekwe et al.^55^. After PCR amplification, the amplicons were resolved on a 1.5% agarose gel to separate and remove residual primers and primer dimers. The bacterial target bands (395 bp) were excised, and DNA was extracted from the gel slices using a PCR Clean-Up Kit (Shanghai Meiji Biomedicals, China). Then, Gel‐purified polymerase chain reaction products were mixed with equal molar and were sequenced on Illumina MiSeq PE300 platform with a paired-end protocol in Shanghai Meiji Biomedical Technology Co., Ltd.

The raw sequencing reads were initially processed using Trimmomatic (v0.38) to quality-filter the raw FASTQ files and assign reads to their respective samples based on unique nucleotide barcodes. Subsequently, FLASH software (v1.2.7) was employed to assemble the paired-end sequences after the removal of primers and nucleotide barcodes. The resulting sequences were then classified into operational taxonomic units (OTUs) with a 97% similarity cutoff using Uparse (v11), during which chimeric sequences were identified and removed using UCHIME (v4.2)^56^. The representative sequences of the OTUs were taxonomically annotated using the SILVA database (v138) and further classified at species, genus, family, order, and phylum levels using the RDP Classifier against the RDP database (v11.5) with a confidence threshold of 0.8^6^.

### Bioinformatical and statistical analyses

Principal coordinate analysis (PCoA) was employed to evaluate the differences in bacterial community composition using Bray-Curtis distances. The analysis of similarity (ANOSIM) was performed to examine the differences among the bacterial community composition among samples from different biofertilizer-treated levels and developmental stages, and confidence intervals for the ANOSIM were estimated from 999 random permutations. Then, we used permutational multivariate analyses of variance (i.e., treatments and developmental stages) with Bray-Curtis dissimilarity on the previously described composition matrices, using the function adonis from the vegan package (v2.6.10) with 999 permutations. Moreover, a multiple range test of the Turkey HSD test by SPSS (v26.0) was used to examine the differences of soil main chemical characteristics, the foliar chemical compounds, microbial community abundances across the samples.

For the taxonomically constitutions of the rhizospheric bacterial community of Tobacco as well as the endogenous ones from roots and leaves sites, the relative abundance at phylum level (> 1%) and genus level (top 30) were calculated and presented by column diagrams. FAPROTAX analysis by FAPROTAX software (v1.2.1) was used to assess differences in ecologically relevant functions of rhizosphere bacteria under biofertilizer treatments^57^. PICRUSt (v2.2.0) was used to predict KEGG functional categories of bacterial functions of bacterial groups dwelled in root and leaf sites^58^. The KEGG level two was kept to present relative abundance of physiological metabolism of endogenous microbial community.

Considering the limitation of the number of replicates, only the bacterial networks in biofertilizer-treated groups were constructed from three niches levels of Tobacco. To visualize the associations in the network, we constructed a correlation matrix by calculating the possible pairwise Spearman’s rank correlations. A valid co-occurrence was considered a statistically robust correlation between species with the Spearman’s correlation coefficient (r) > 0.65 and the *P*-value < 0.01. The P-values were adjusted by a multiple testing correction using the BenjaminieHochberg method to reduce the chances of obtaining false-positive results^59^. The nodes in the constructed networks represent bacterial genera and edges represent strong and significant correlations between genera. The network topological properties (e.g., Average path length, modularity index, Average clustering coefficient) were calculated for both observed and random networks using Gephi (v9.0)^60^. The keystone taxa were determined by analyzing intra-module connectivity (Zi) and inter-module connectivity (Pi), including classifications such as connectors (Zi < 2.5&Pi > 0.62), module hubs (Zi > 2.5&Pi < 0.62), and network hubs (Zi > 2.5&Pi > 0.62)^34^. They played a crucial role in maintaining the robustness of the co-occurrence networks, meaning they occupy important ecological positions^24^. Here, we calculated the correlations of modules in rhizospheric bacterial network and soil properties, as well as modules in foliar network and leaf quality properties, and presented the results in the form of heatmaps. And the relative abundance of bacterial community of the network modules in the treatments were summarized. Furthermore, we selected the top 100 most abundant soil bacterial genera and foliar bacterial genera, and employed Spearman’s rank analysis to evaluate their associations with soil physicochemical properties and tobacco leaf chemical components, respectively. The correlation values were retained and visualized in a heatmap, with a significance level set at *P*-value < 0.05.

## Electronic supplementary material

Below is the link to the electronic supplementary material.

Supplementary Material 1.

## Data Availability

Sequence data that support the findings of this study have been deposited in the NCBI Sequence Read Archive (SRA) repository with accession number PRJNA1164948.
