# Supplementary material for: Impact of fermented rapeseed cake mixed Bacillus velezensis on the bacterial community structure and cultivation of tobacco cultivar K326
Source: Sci Rep. 2025 Jul 10;15:24818. doi: 10.1038/s41598-025-08400-9 (PMC12246417; doi:10.1038/s41598-025-08400-9)
Supplement: Supplementary file 1 — Supplementary Material 1. [file 41598_2025_8400_MOESM1_ESM.docx]

Table S1 Agricultural management measures of fertilizer times and use levels for tobacco K326.

| fertilizers | use level (kg/mu) | fertilization timing after transplanting |
| --- | --- | --- |
| compound fertilizer  (N-P2O5-K2O: 20-9-0) | 2 | when transplanted the seedling |
| compound fertilizer  (N-P2O5-K2O: 20-9-0) | 3.5 | within the first week |
| H5KMgO4S fertilizer | 2.5 |  |
| KNO₃ fertilizer | 10 | within the second week |
| H5KMgO4S fertilizer | 2.5 |  |
| KNO₃ fertilizer | 15 | within the third week |
| K₂SO₄ fertilizer | 20 |  |

Table S2 The main constituents of the *Bacillus velezensis* bacterial biofertilizer applied as the base fertilizer.

|  | Total N | Total nutrition  (N-P2O5-K2O) | Organic matter | pH | *Bacillus velezensis* |
| --- | --- | --- | --- | --- | --- |
| The tested biofertilizer | >5.2% | >15% | >50% | 6.8 | >1.0 CFU/mL |

Table S3 The standard content levels of four chemical substances in middle Tobacco leaves for making high-quality tobacco products.

| levels (%) | middle leaves |
| --- | --- |
| TS (Total Sugar) | 18%-24% |
| TN (Total N) | 1.5%-3.5% |
| TK (Total K) | > 2% |
| NT (Nicotine) | ~ 2.5% |

Table S4 The bacterial genera with their relative abundance changes lined with BOF treated levels in soil, root and leaves of tobacco k326.

| habitats | genera | Control | B30 | B60 | B90 | Control | B30 | B60 | B90 |
| --- | --- | --- | --- | --- | --- | --- | --- | --- | --- |
|  |  | 45d | | | | 90d | | | |
| rhizosphere | Marmoricola | 1.45c | 2.26b | 3.96ab | 5.56a | 2.36b | 2.92ab | 3.10c | 3.29c |
|  | Sphingomonas | 3.09b | 3.93b | 4.40b | 6.55a | 1.27a | 1.26a | 1.47a | 1.91a |
|  | Bacillus | 2.46c | 2.75c | 3.55b | 4.94a | 1.48b | 2.24ab | 2.43a | 2.48a |
|  | Nocardioides | 1.96b | 1.39b | 3.92a | 3.32a | 1.78b | 1.54b | 1.62b | 2.05a |
|  | Lysobacter | 0.67b | 1.07a | 1.19a | 1.82a | - | - | - | - |
| root | Flavobacterium | 6.747c | 9.087b | 10.856ab | 14.704a | - | - | - | - |
|  | Ensifer | 2.567b | 2.508b | 5.25a | 5.694a | 2.56b | 4.21a | 3.86a | 3.23a |
|  | A.-N.-P.-R. | 0.968b | 1.102b | 1.968a | 2.508a | 3.173b | 3.604a | 4.378a | 3.934a |
|  | Sphingomonas | 0.883c | 2.343b | 4.69a | 3.96a | 1.54b | 2.056ab | 2.34a | 2.46a |
|  | Pseudomonas | 1.416b | 2.02b | 2.57b | 3.34a | 1.86b | 2.14b | 2.36b | 4.21a |
|  | Sphingobium | 0.996b | 1.411b | 2.776a | 1.99ab | - | - | - | - |
|  | Lysinimonas | - | - | - | - | 1.118b | 1.472b | 1.441b | 2.255a |
| leaves | Sphingomonas |  |  |  |  | 18.04b | 13.477c | 26.896a | 21.438a |
|  | Pseudomonas | 12.84c | 15.87c | 20.56b | 28.21a |  |  |  |  |
|  | Chryseobacterium | - | - | - | - | 16.46b | 24.67a | 26.7a | 30.34a |
|  | Bacillus | 3.547b | 4.619b | 6.408a | 6.196a | - | - | - | - |
|  | Corynebacterium | 1.57c | 2.44b | 2.93ab | 3.83a | - | - | - | - |
|  | B.-C.-P. | 0.63ab | 1.08a | 1.03a | 1.20a | - | - | - | - |

notes: “-” meant the changes tendency of the bacteria relative abundance not complying with biofertilizer addition levels.

“A.-N.-P.-R.” indicated the bacterial genus of Allorhizobium-Neorhizobium-Pararhizobium-Rhizobium

“B.-C.-P.” indicated the bacterial genus of Burkholderia-Caballeronia-Paraburkholderia

Table S5 Key topological features of rhizosphere and endogenous bacterial networks.

|  | topological features | soil community | root community | leaf community |
| --- | --- | --- | --- | --- |
| empirical networks | Nodes | 375 | 238 | 401 |
|  | Edges | 1803 | 1016 | 3104 |
|  | Average degree (AD) | 9.616 | 8.538 | 15.481 |
|  | Average path length (APL) | 3.731 | 5.105 | 4.211 |
|  | Module index (MI) | 0.463 | 0.549 | 0.512 |
|  | Average clustering coefficient (ACC) | 0.368 | 0.537 | 0.704 |
|  | Diameter | 11 | 15 | 12 |
|  | Intensity | 0.026 | 0.036 | 0.039 |
| random networks | ACC | 0.060±0.005 | 0.117±0.014 | 0.157±0.018 |
|  | APL | 2.964±0.020 | 2.925±0.074 | 2.688±0.050 |
|  | modularity | 0.16±0.005 | 0.156±0.006 | 0.14±0.005 |

Table S6 The summary of bacterial genus (that is, nodes) in each module from each co-occurrence network.

| habitats | module | Genera counts belonging to phyla levels |
| --- | --- | --- |
| Soil | M#3 | Firmicutes (16); Actinobacteriota (13); Proteobacteria (13); Acidobacteriota (11); Chloroflexi (5); Planctomycetota (5); Verrucomicrobiota (5); Patescibacteria (2); Bacteroidota (1); Sumerlaeota (1); WS2 (1) |
|  | M#1 | Proteobacteria (20); Acidobacteriota (11); Chloroflexi (5); Actinobacteriota (4); Methylomirabilota (3); Latescibacterota (2); Myxococcota (2); Planctomycetota (1); Verrucomicrobiota (1); Bdellovibrionota (1); Desulfobacterota (1); Entotheonellaeota (1); GAL15 (1); Gemmatimonadota (1); NB1-j (1); Nitrospirota (1) |
|  | M#4 | Proteobacteria (23); Actinobacteriota (15); Chloroflexi (5); Acidobacteriota (4); Myxococcota (3); Bacteroidota (3); Planctomycetota (1); Entotheonellaeota (1); Gemmatimonadota (1); Firmicutes (1); d_Bacteria (1) |
|  | M#0 | Proteobacteria (19); Actinobacteriota (11); Bacteroidota (10); Myxococcota (3); Firmicutes (3); Acidobacteriota (2); Patescibacteria (2); Gemmatimonadota (1) |
|  | M#5 | Proteobacteria (16); Actinobacteriota (13); Firmicutes (4); Bacteroidota (2); Myxococcota (2); Acidobacteriota (2); Chloroflexi (2); Desulfobacterota (2); Nitrospirota (1); Dadabacteria (1); MBNT15 (1); RCP2-54 (1); Sva0485 (1) |
|  | M#10 | Proteobacteria (13); Actinobacteriota (7); Acidobacteriota (6); Chloroflexi (4); Gemmatimonadota (4); Firmicutes (3); Bacteroidota (3); Myxococcota (1); Planctomycetota (1); Cyanobacteria (1) |
| Root | M#0 | Proteobacteria (29); Actinobacteriota (13); Bacteroidota (4); Firmicutes (2); Patescibacteria (1); Spirochaetota (1); d_Bacteria (1) |
|  | M#2 | Proteobacteria (25); Actinobacteriota (11); Bacteroidota (2); Acidobacteriota (1); Bdellovibrionota (1); Verrucomicrobiota (1 |
|  | M#5 | Firmicutes (15); Bacteroidota (9); Actinobacteriota (7); Patescibacteria (3) |
|  | M#9 | Proteobacteria (17); Actinobacteriota (3); Myxococcota (3); Bacteroidota (2); Acidobacteriota (2); Firmicutes (1); Bdellovibrionota (1); Armatimonadota (1); Gemmatimonadota (1); Nitrospirota (1 |
|  | M#13 | Proteobacteria (13); Actinobacteriota (7); Firmicutes (4); Myxococcota (2); Chloroflexi (2); Bdellovibrionota (1); Gemmatimonadota (1) |
|  | M#7 | Proteobacteria (13); Actinobacteriota (2); Myxococcota (1 |
| Leaf | M#2 | Proteobacteria (50); Actinobacteriota (17); Firmicutes (15); Bacteroidota (13); Methylomirabilota (4); Chloroflexi (2); d_Bacteria (1) |
|  | M#3 | Actinobacteriota (31); Proteobacteria (20); Firmicutes (11); Bacteroidota (4); Chloroflexi (3); Bdellovibrionota (2); Myxococcota (2); Acidobacteriota (1); Gemmatimonadota (1); NB1-j (1); Planctomycetota (1) |
|  | M#0 | Proteobacteria (30); Actinobacteriota (19); Firmicutes (8); Myxococcota (3); Bacteroidota (2); Acidobacteriota (2); Chloroflexi (1); Bdellovibrionota (1); Gemmatimonadota (1); Armatimonadota (1); Nitrospirota (1) |
|  | M#8 | Firmicutes (16); Proteobacteria (14); Actinobacteriota (14); Bacteroidota (4); Chloroflexi (3); Acidobacteriota (1); Caldatribacteriota (1); Synergistota (1); Verrucomicrobiota (1); Thermotogota (1) |
|  | M#6 | Proteobacteria (11); Firmicutes (10); Actinobacteriota (10); Bacteroidota (4); Chloroflexi (4); Acidobacteriota (3); Synergistota (1); Verrucomicrobiota (1); Myxococcota (1); Deinococcota (1); Desulfobacterota (1); Entotheonellaeota (1) |
|  | M#5 | Proteobacteria (9); Actinobacteriota (6); Firmicutes (5); Myxococcota (3); Verrucomicrobiota (1); Nitrospirota (1) |

Table S7 the keystone species (Connectors, Module hubs, etc.) of three co-occurrence networks

| type | phylum | genus | abundance | phylum | genus | abundance | phylum | genus | abundance |
| --- | --- | --- | --- | --- | --- | --- | --- | --- | --- |
|  | rhizosphere | | | root | | | leaf | | |
| Connectors | Actinobacteriota | 67-14 (f) | 13961 | Actinobacteriota | Streptomyces | 43690 | Actinobacteriota | Nocardioides | 3810 |
|  |  | Nocardioides | 11357 |  | Agromyces | 1003 |  | Cutibacterium | 1730 |
|  |  | Micromonospora | 9199 |  | Solirubrobacter | 969 |  | MB-A2-108 (c) | 1132 |
|  |  | Mycobacterium | 6092 |  | 67-14 (f) | 1108 |  | Thermobifida | 825 |
|  |  | Ilumatobacteraceae (f) | 2972 | Bdellovibrionota | Bdellovibrio | 3231 |  | Lysinimonas | 495 |
|  |  | Kribbella | 1552 |  | Peredibacter | 702 |  | Curtobacterium | 488 |
|  |  | Hamadaea | 1159 | Firmicutes | Bacillus | 4494 |  | Terrabacter | 461 |
|  | Chloroflexi | JG30-KF-CM45 (f) | 7328 | Nitrospirota | Nitrospira | 680 | Chloroflexi | Roseiflexaceae (f) | 488 |
|  |  | S085 (o) | 2270 | Proteobacteria | Sphingomonas | 23808 | Firmicutes | Weissella | 7063 |
|  |  | C0119 (o) | 1933 |  | Pseudomonas | 13790 |  | Lactococcus | 1245 |
|  |  | JG30-KF-CM66 (c) | 1853 |  | Sphingopyxis | 11579 |  | Dorea | 910 |
|  | Desulfobacterota | Desulfobacterota (p) | 1871 |  | Neorhizobium | 8509 |  | Peptostreptococcales-Tissierellales (o) | 554 |
|  | Firmicutes | Paenibacillus | 1115 |  | Alcaligenaceae (f) | 4668 | Proteobacteria | Massilia | 39967 |
|  |  | Hydrogenispora | 1089 |  | Sphingomonadaceae (f) | 3514 |  | Stenotrophomonas | 22721 |
|  | Methylomirabilota | Rokubacteriales (o) | 9593 |  | Enterobacter | 3253 |  | Bordetella | 9993 |
|  | Proteobacteria | Xanthobacteraceae (f) | 15318 |  | Phreatobacter | 2233 |  | Xanthomonadaceae (f) | 2074 |
|  |  | Defluviicoccus | 5761 |  | Proteobacteria (p) | 1970 |  | Gammaproteobacteria (c) | 1484 |
|  |  | Ellin6067 | 2564 |  | Pseudolabrys | 1323 |  | Steroidobacter | 557 |
|  |  | Sphingobium | 1169 |  | Altererythrobacter | 1157 |  | Lysobacter | 546 |
|  |  | Steroidobacteraceae (f) | 1072 |  | Ferrovibrio | 887 |  | Rhodocyclaceae (f) | 509 |
|  |  | others |  |  | others |  |  | others |  |
| Module hubs | Acidobacteriota | Candidatus_Solibacter | 1559 | Proteobacteria | Xanthobacteraceae (f) | 2279 |  |  |  |
|  | Proteobacteria | Methyloligellaceae (f) | 4957 | Actinobacteriota | Nocardioides | 12391 |  |  |  |
|  | Myxococcota | Haliangium | 2784 |  |  |  |  |  |  |


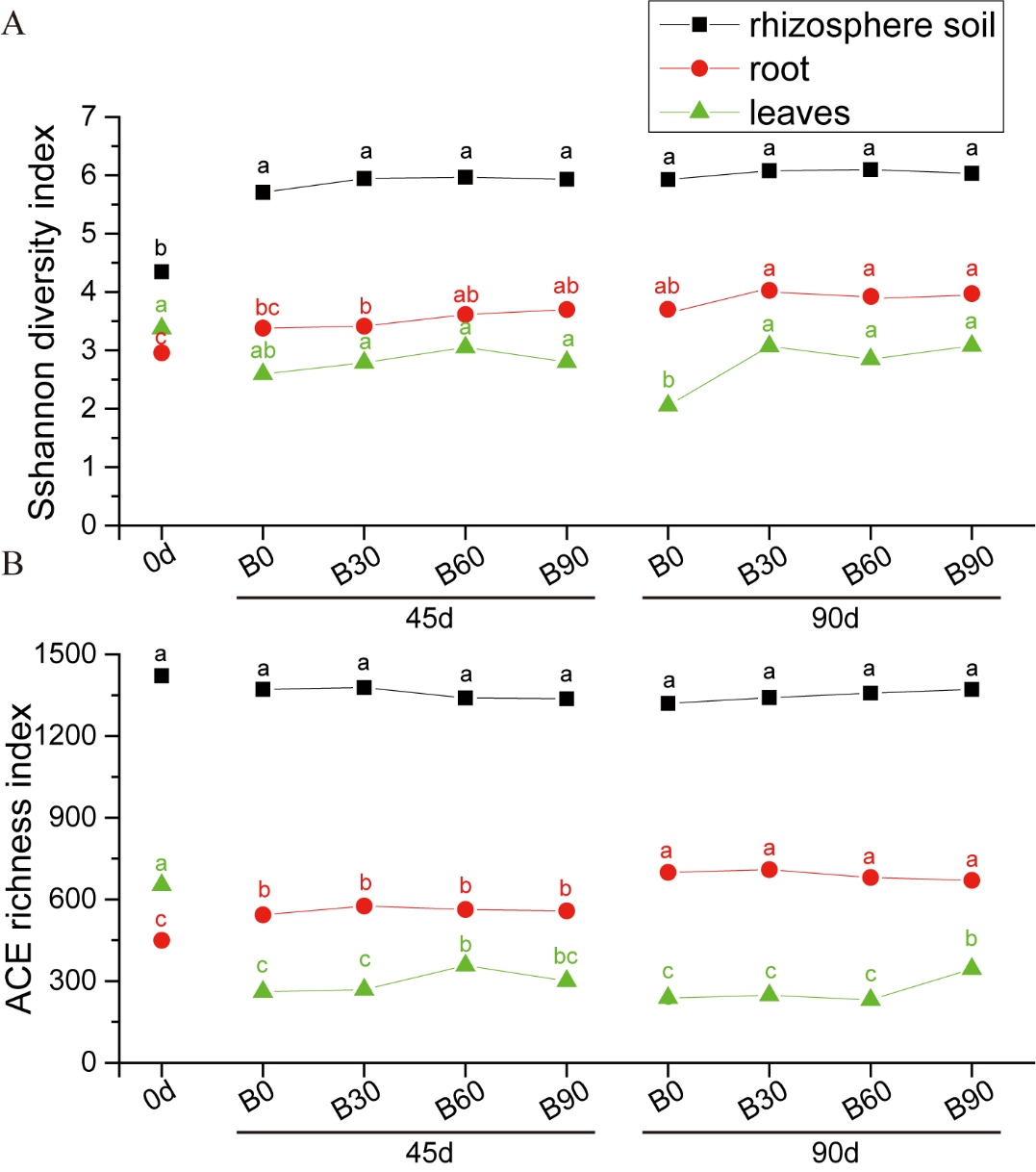


Fig. S1 Changes of Shannon diversity index (A) and ACE richness index (B) of three bacterial community after transplanting days of 0d, 50d, 100d. S, rhizosphere soil; R, root; L, leaf.


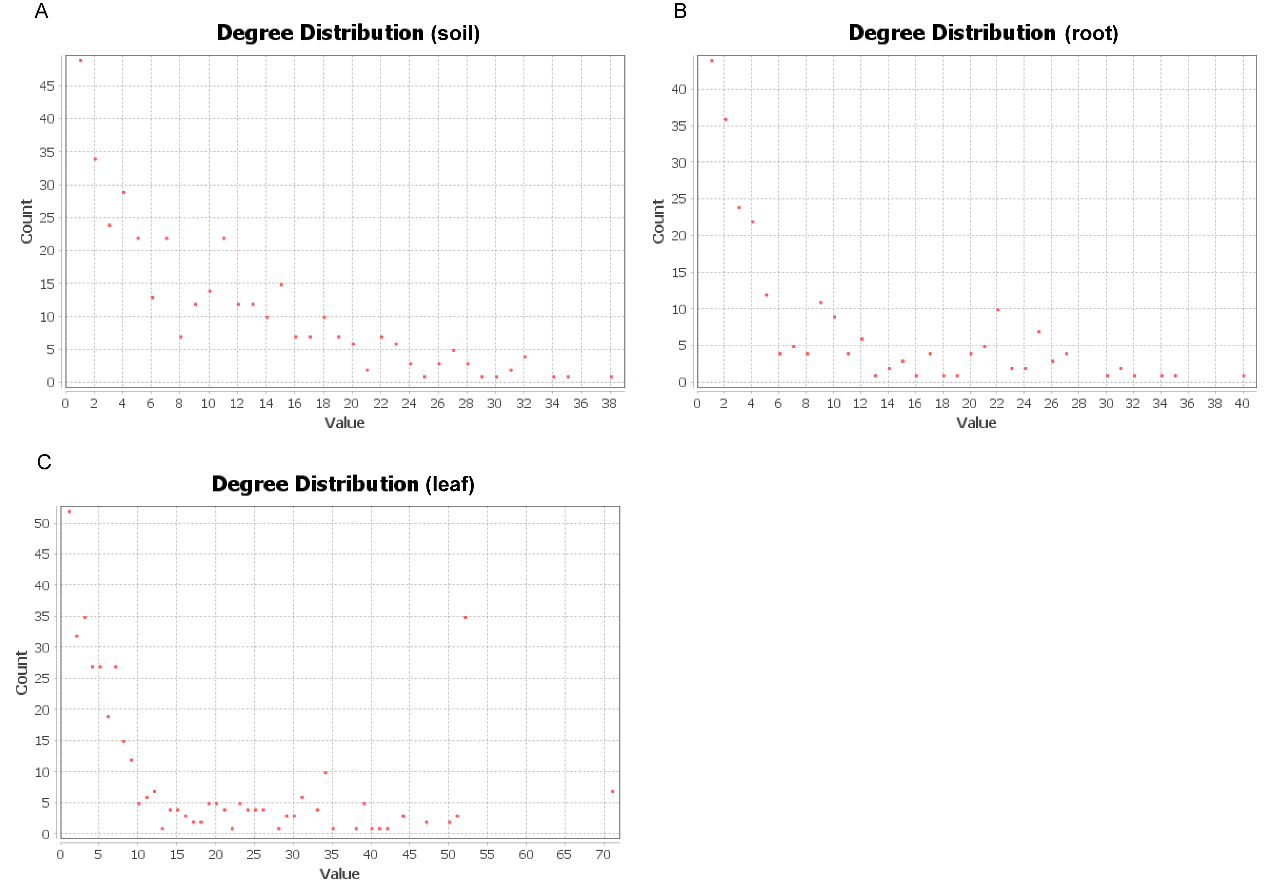


Fig. S2 The network degree distribution patterns of bacteria in rhizosphere, root and leaves of Tobacco.


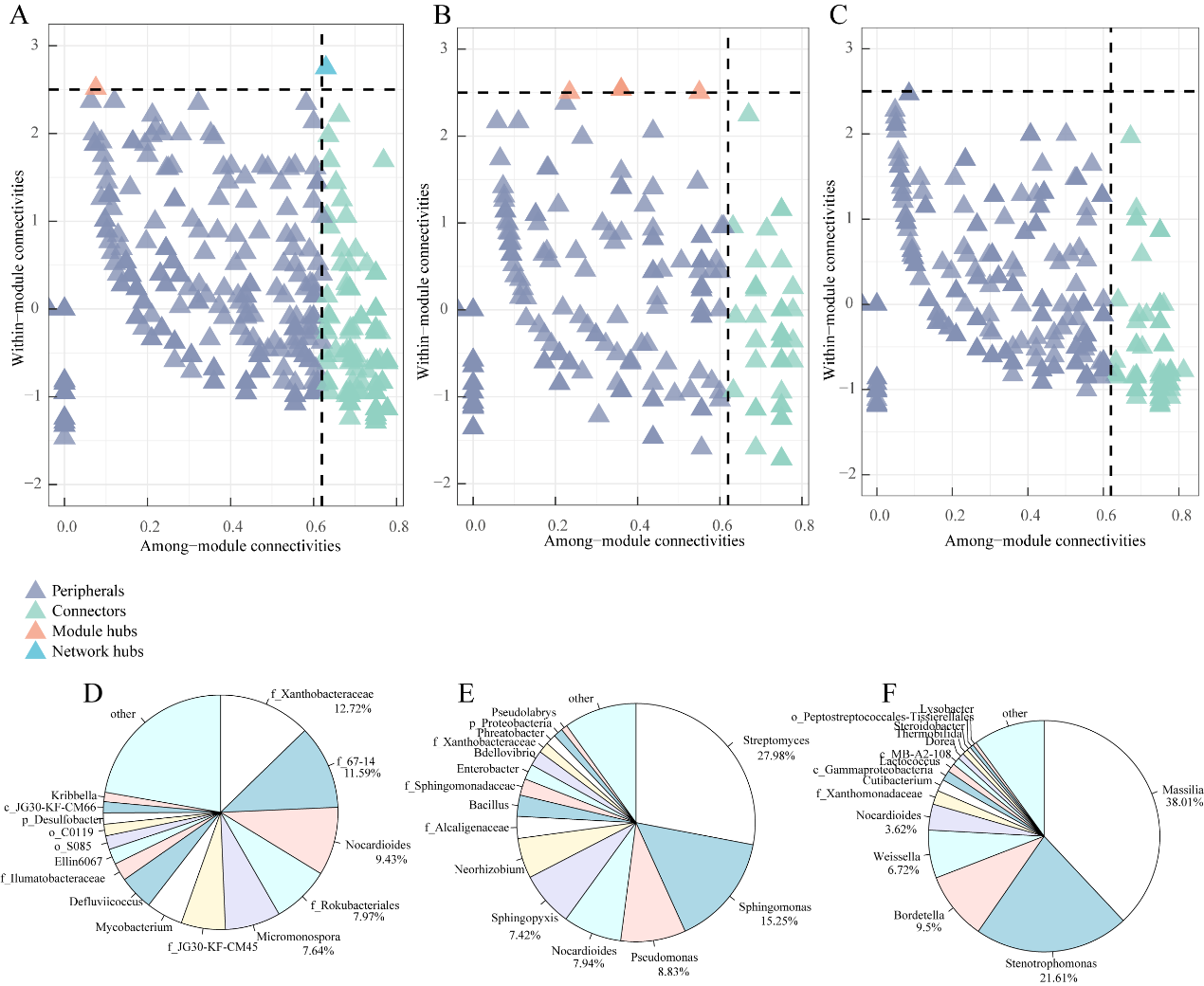


Fig. S3 Co-occurrence networks of bacteria (genus level) from tobacco rhizosphere (A), root (B), leaves (C) habitats based on Spearman correlation analysis. Zi-Pi plots of three habitats (D, rhizosphere; E, root; F, leaves) based on genus topological roles in bacterial networks. The threshold values of Zi and Pi for categorizing OTUs were 2.5 and 0.62, respectively.


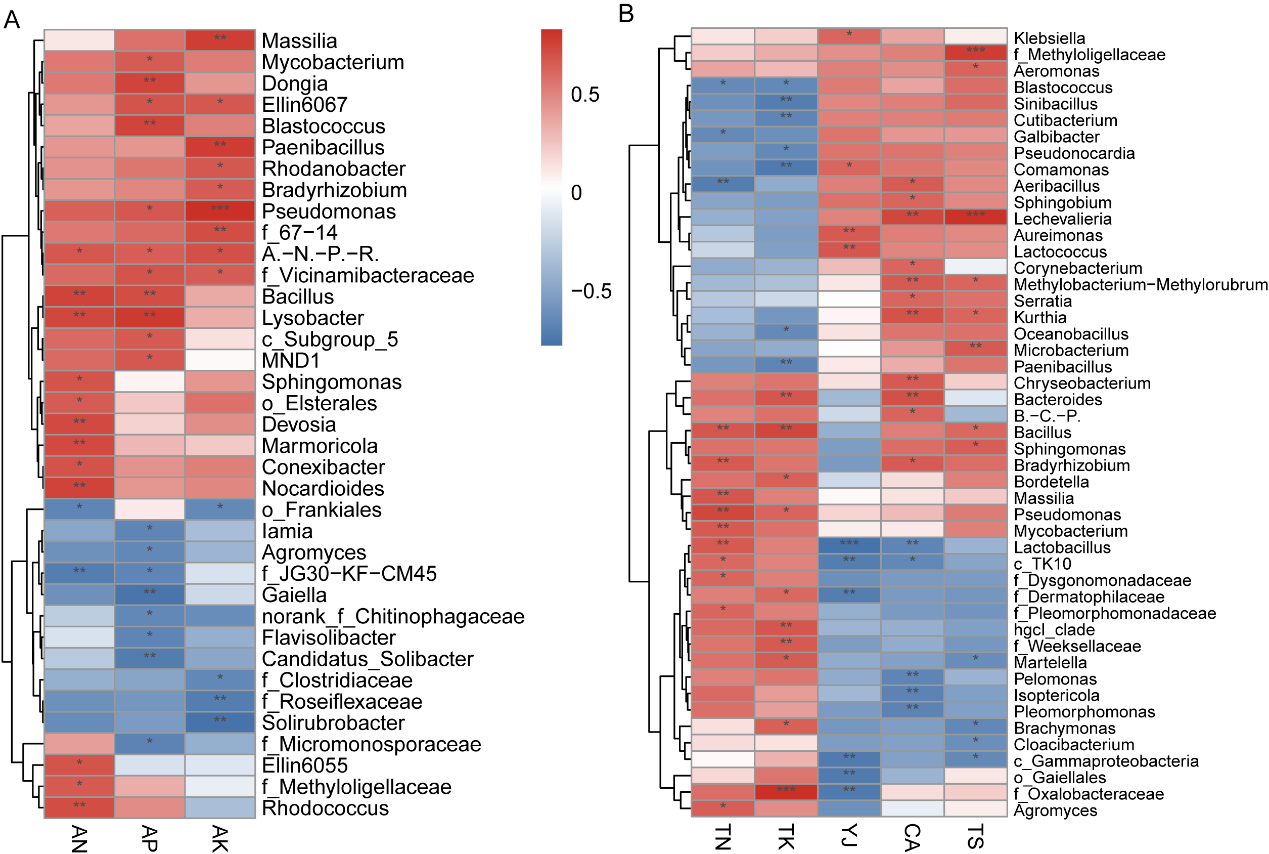


Fig. S4 The associations between the top 100 bacterial genera (in rhizosphere soil and tobacco leaf microbiota) and soil physicochemical properties (A)/leaf chemical compositions (B) based on Spearman’s rank correlation analysis. All p-values were adjusted using the Benjamini-Hochberg (BH) false discovery rate correction. Statistically significant correlations (*p* < 0.05) were subsequently visualized using hierarchical clustering heatmaps.

Soil physicochemical properties were AN (alkali-hydrolyzed nitrogen), AP (available phosphate), AK (available potassium). Leaf chemical compositions were TN (total nitrogen), TK (Total K), YJ (nicotine), CA (chlorogenic acid), TS (total sugar).

“A.-N.-P.-R.” indicated the bacterial genus of Allorhizobium-Neorhizobium-Pararhizobium-Rhizobium

“B.-C.-P.” indicated the bacterial genus of Burkholderia-Caballeronia-Paraburkholderia
